# The Effectiveness of Supportive Psychotherapy on the Anxiety and Depression Experienced by Patients Receiving Fiberoptic Bronchoscope

**DOI:** 10.3389/fpsyg.2022.960049

**Published:** 2022-07-25

**Authors:** Fengjuan Ren, Dan Ruan, Weilin Hu, Yan Xiong, Yuwan Wu, Siyu Huang

**Affiliations:** Department of Respiratory and Critical Care Medicine, Wuhan Wuchang Hospital, Wuhan, China

**Keywords:** supportive psychotherapy, fiberoptic bronchoscope, anxiety, depression, mental health, satisfaction

## Abstract

**Objectives:**

As the largest cohort of healthcare workers and nurses can practice as psychotherapists to integrate the psychotherapeutic interventions as part of routine care. The present study aims to evaluate the effectiveness of supportive psychotherapy (SPT) on patients who had been scheduled to undergo a fiberoptic bronchoscopy (FOB) procedure.

**Methods:**

This study retrospectively analyzed 92 patients who underwent FOB, which was divided into the SPT group and usual-care group based on whether patients were given SPT interventions or not. The Patient Health Questionnaire-9 (PHQ-9) and Hospital Anxiety and Depression Scale (HADS) were used to determine the severity of depression and anxiety, as well as the 36-Item Short-Form Health Survey questionnaire (SF-36) to evaluate the health-related quality of life (HRQoL). Moreover, the patients' satisfaction was assessed based on the Likert 5-Point Scale.

**Results:**

The baseline status of anxiety, depression, and HRQoL in patients was similar in the SPT group and the usual-care group with no significant difference. Both PHQ-9 score and HADS-D score in the SPT group after intervention were lower than that in the usual-care group, accompanied by the deceased HADS-A subscale. Moreover, the improved HRQoL was found in the patients undergoing FOB after SPT interventions as compared to those receiving conventional nursing care using the SF-36 score. Additionally, the patient satisfaction in the SPT group was higher than in the usual-care group.

**Conclusions:**

The study demonstrated that anxiety and depression, as negative emotions, can be reduced by supportive psychotherapy in patients receiving FOB with improved mental health and satisfaction with nursing care.

## Introduction

As difficulties in airway management contribute significantly to severe anesthesia-related morbidity and mortality, securing the airway in a safe fashion remains a concern for anesthetists (Li et al., 2021). During the procedure, awake intubation was reported to provide patients' safety *via* keeping airway patency, maintaining gas exchange, and protecting against aspiration, thus being a recommended option for the management of difficult airways (Jiang et al., 2018; Utada et al., 2022). The fiberoptic bronchoscope (FOB) could be manually manipulated and seen around corners, which, therefore, is a common choice for awake intubation (Sachdev et al., 2022). As demonstrated by a previous study, sputum suction and lavage by FOB promote the treatment effect in patients with severe pneumonia with the improvement of blood oxygen and a decreased inflammatory reaction state (Han et al., 2019). In addition, although FOB has been regarded as a “gold standard” tool for managing difficult airways, it could result in several related complications, such as accidental extubating, perioperative hemorrhage, tracheal ring rupture, lesions of the tracheal wall, and abnormal insertion of the cannula (Cabrini et al., 2006), which could finally result in adverse emotions, such as anxiety and depression (Shyam et al., 2017; Opartpunyasarn et al., 2022).

Psychotherapy is not a new form of care performed by nurses, and nurses, as the largest cohort of healthcare workers, are suitable candidates for therapeutic counseling work as they possess relevant background knowledge on disease presentations, treatment plans, and side effects, as well as first-hand patient experiences (Tay et al., 2018; Malakian et al., 2022). The negative emotions, such as fear, anxiety, and depression in most patients in the early stage of admission, could be alleviated through good psychological care (Lin et al., 2021). Supportive psychotherapy (SPT) as a nonspecific, nondirective treatment is possibly the most ubiquitously used psychotherapy, which required no formal or special training (Grover et al., 2020). As reported by previous studies, SPT performed by nurses is an “inferior” therapy for several diseases, such as chronic back pain (Rutledge et al., 2018), hematologic malignancies (Koehler et al., 2022), body dysmorphic disorder (Weingarden et al., 2021), obesity (Juchacz et al., 2021), and lung cancer (Peng et al., 2019), can offer significant benefits *via* improving effectively functional impairment, depression severity and quality of life. Although SPT for adults may alleviate psychological distress (Koehler et al., 2022), little is known about the practice of nursing when providing this form of care to patients undergoing FOB. Therefore, we enrolled two groups of patients, namely the SPT group and the usual-care group, to investigate the effect of SPT on the negative emotions, mental health, and satisfaction of these undergoing FOB.

## Methods

### Participants

Ninety-two adult patients of either sex requiring general anesthesia with endotracheal intubation were enrolled, which were in American Society of Anesthesiologists (ASA) class I/II (Sankar et al., 2014) and in modified Mallampati classification I/II (Islam et al., 2015). All patients were intubated by experienced anesthesiologists using a FOB (MAF-TM Portable Bronchoscope; Olympus, Tokyo, Japan) according to the previous study (Luo et al., 2022). All patients consented to participate and provided written informed consent.

### Exclusion Criteria

Exclusion criteria were as follows: (1) patients refusing to participate; (2) patients suffering from respiratory illnesses, such as bronchial asthma or a history of airway hyperreactivity; (3) patients with known allergies to drugs; (4) patients undergoing emergency procedures, obstetric procedures, or on beta-blockers; (5) patients with cervical spine disease, lose or missing incisors, preoperative hoarseness, hypertension, and abnormalities of heart, brain, liver, lung, kidney, and coagulation functions; (6) patients unable to provide their consent. The participants were randomly divided into the SPT group and usual-care group, and they firstly finished the following questionnaires to reflect their status of anxiety, depression, and mental health at baseline. The subjects in the usual-care group received conventional nursing care mainly *via* informing the patient of general preparation and precautions during FOB, as well as providing the measurement of ECG, non-invasive respiration, and pulse oximetry.

### SPT Interventions

The patients in SPT group received SPT intervention before, during and after FOB in addition to conventional nursing care, including the following items: (1) a suitable psychological nursing approach for patients was chosen according to the illness and psychological state of patients to establish a good doctor-patient relationship and improve patient compliance with FOB; (2) the methods, advantages and precautions of FOB should be explained in detail so as to reduce the psychological burden of patients; (3) for anxious patients, psychological counseling should be timely given with the maintenance of good communication with family members; (4) during the examination, assisting the patients lie in the correct position, asking if there was any discomfort, and cooperating with the doctors were needed; (5) after the examination, the patient was instructed to fast for 4–6 h and to avoid eating irritating food within 48 h accompanying by the advisement of talking as little as possible and avoiding vigorous coughing; and (6) an explanation of discomforts should be given to the patients, including pain and discomfort in the throat and nasal, cough, hoarseness, etc., which would be relieved after adequate rest.

### Patient Health Questionnaire-9

The Patient Health Questionnaire-9 (PHQ-9) is a nine-item questionnaire on a 0–3 scale, including anhedonia, depressed mood, sleep disturbance, fatigue, appetite changes, low self-esteem, concentration problems, psychomotor disturbances, and suicidal ideation, designed for assessing the depression (Levis et al., 2019; Patel et al., 2019). Total scores of PHQ-9 range from 0 to 27.

### Hospital Anxiety and Depression Scale

The Hospital Anxiety and Depression Scale (HADS) has two subscales evaluating anxiety (HADS-A) and depression (HADS-D), and each was composed of a 7- item score from 0 to 3 with a maximum score of 21 (Akgor et al., 2021). The scores ranging from 0 to 7 showed healthy patients, 8–11 indicated borderline for depression/anxiety, and ≥ 11 represented severe depression/anxiety.

### 36-Item Short-Form Health Survey Questionnaire

The 36-Item Short-Form Health Survey questionnaire (SF-36) is one of the most generally used instruments for evaluating the health-related quality of life (HRQoL) (Lins and Carvalho, 2016), which includes eight multiple-item subscales consisting of 36 questions: physical functioning (10 items), general health (5 items), role physical (4 items), bodily pain (2 items), social functioning (2 items), vitality (4 items), role emotional (3 items), and mental health (5 items). The total score on each SF-36 subscale ranges between 0 and 100.

### Likert 5-Point Scale Measurement

The satisfaction sections were measured using the Likert 5-Point Scale (Zhi et al., 2021), wherein 1 = completely dissatisfied, 2 = dissatisfied, 3 = partially satisfied, 4 = satisfied, and 5 = completely satisfied. The higher the total score means the higher the satisfaction.

### Statistics Analysis

Statistical analyses were done with GraphPad prism. Continuous variables and categorical variables were reported as mean ± *SD* and count/proportion, respectively. Comparisons of proportions were made using χ^2^ and Fisher's exact tests, and continuous variables Student's *t*-tests, with the two-sided *P*-values less than 0.05 as statistically significant.

## Results

### The Demographic Profile of the Two Groups

The baseline characteristic of patients from the SPT group (*n* = 47) and the usual-care group (*n* = 45) was shown in Table 1. There was no significant difference between the two groups, regarding to age (*P* = 0.608), gender (*P* = 0.297), weight (*P* = 0.928), ASA status (*P* = 0.307) and Mallampati classification (*P* = 0.417).

**Table 1:** The demographic profile of the two groups.

**Parameters**	**SPT group** **(*n* = 47)**	**Usual-care group** **(*n* = 45)**	**χ^2^/t**	***P***
Gender				
Male	21	25		
Female	26	20	1.807	0.297
Age (years)	49.79 ± 12.20	48.47 ± 12.39	0.515	0.608
Weight (kg)	65.50 ± 10.17	65.69 ± 10.32	0.090	0.928
ASA status				
I	31	25		
II	16	20	1.044	0.307
Mallampati classification				
I	29	24		
II	18	21	0.659	0.417

*SPT, Supportive psychotherapy; ASA, American Society of Anesthesiologists*.

### The Reduced Depressive and Anxious Symptoms in Patients Using FOB After SPT Interventions

**Figure 1:**
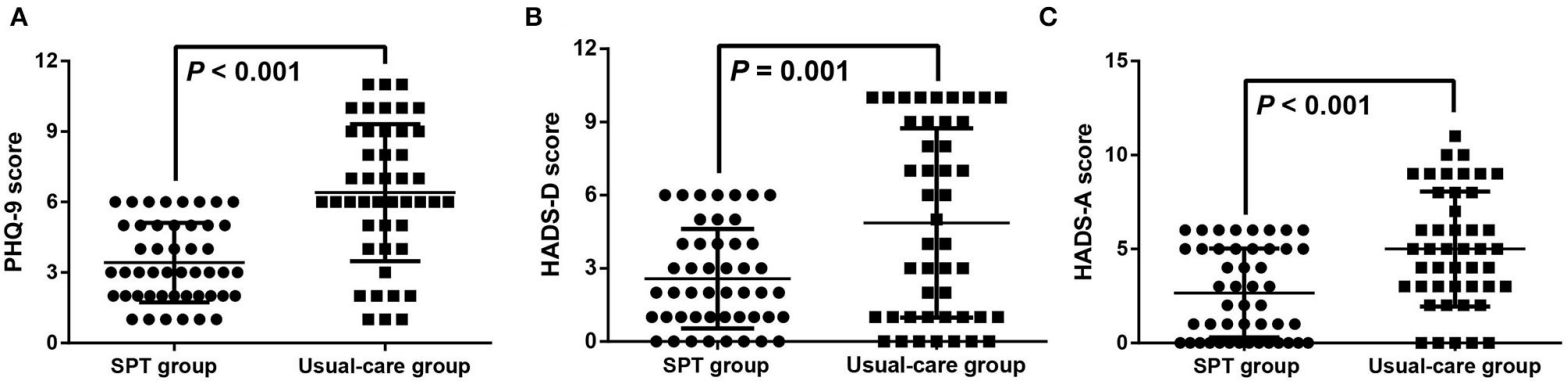
The reduced depressive and anxiety symptoms in patients undergoing fiberoptic bronchoscope (FOB) after supportive psychotherapy (SPT). The depressive symptoms were evaluated by the Patient Health Questionnaire-9 (PHQ-9) score **(A)** and Depression subscale of the Hospital Anxiety and Depression Scale (HADS-D) score **(B)**, and the anxious status was evaluated by the Anxiety subscale of the HADS-A score **(C)**.

As shown in Table 2, the baseline depressive status in patients was similar in the SPT group and the usual-care group with no significant difference in terms of PHQ-9 score (4.98 ± 2.92 vs. 5.64 ± 2.72, *P* = 0.262) and HADS-D score (3.91 ± 2.95 vs. 4.11 ± 3.2, *P* = 0.76). Both PHQ-9 score (3.43 ± 1.69 vs. 6.4 ± 2.91, *P* < 0.001), and HADS-D score (3.04 ± 2.3 vs. 4.87 ± 3.88, *P* = 0.001) in the SPT group after the intervention was lower than that in the usual-care group (Table 2 and Figures 1A,B). Furthermore, the patients after treated with SPT was showed the attenuated depressive symptoms with the lower scores of PHQ-9 (3.43 ± 1.69 vs. 4.98 ± 2.92, *P* = 0.002) and HADS-D (3.04 ± 2.3 vs. 3.91 ± 2.95, *P* = 0.012) than that at baseline (Table 2).

**Table 2:** Comparison of the depressive and anxious symptoms in patients undergoing fiberoptic bronchoscope (FOB).

**Parameters**	**SPT group** **(*n* = 47)**	**Usual-care group** **(*n* = 45)**	**t**	***P***
PHQ-9 score				
Baseline	4.98 ± 2.92	5.64 ± 2.72	1.129	0.262
After intervention	3.43 ± 1.69	6.40 ± 2.91	6.025	<0.001
t	3.154	1.272		
*P*	0.002	0.207		
HADS-D score				
Baseline	3.91 ± 2.95	4.11 ± 3.20	0.306	0.760
After intervention	3.04 ± 2.30	4.87 ± 3.88	3.566	0.001
t	2.563	1.007		
*P*	0.012	0.317		
HADS-A score				
Baseline	4.21 ± 3.06	4.64 ± 2.68	0.718	0.475
After intervention	2.66 ± 2.37	5.00 ± 3.06	4.111	<0.001
t	2.748	0.586		
*P*	0.007	0.559		

*SPT, Supportive psychotherapy; PHQ-9, Patient Health Questionnaire-9; HADS-D, Depression subscale of the Hospital Anxiety and Depression Scale; HADS-A, Anxiety subscale of the Hospital Anxiety and Depression Scale*.

### The Alleviated Anxious Status in Patients Undergoing FOB After SPT Interventions

According to the HADS-A subscale (Table 2), the patients in the SPT group and the usual-care group demonstrated no statistical difference in anxious status at the beginning (4.21 ± 3.06 vs. 4.64 ± 2.68, *P* = 0.475). After SPT interventions, a higher HADS-A score was revealed in the usual-care group than in the SPT group (5 ± 3.06 vs. 2.66 ± 2.37, *P* < 0.001, Figure 1C). Moreover, the decreased HADS-A score in the SPT group after SPT interventions was displayed as compared with the baseline (2.66 ± 2.37 vs. 4.21 ± 3.06, *P* = 0.007). Moreover, none of the patients had severe anxiety and depression in both groups at baseline and after the intervention, and the number of healthy patients and those who had mild anxiety and depression has been demonstrated in Table 3.

**Table 3:** The number of patients had different degree of anxiety and depression.

	**SPT (*n* = 47)**		**Usual-care group (*n* = 45)**	
	**0**~**7**	**8**~**11**	**0**~**7**	**8**~**11**
HADS-D				
Baseline	40	7	33	12
After intervention	47	0	30	15
HADS-A				
Baseline	38	9	37	8
After intervention	47	0	33	12

*HADS-D, Depression subscale of the Hospital Anxiety and Depression Scale; HADS-A, Anxiety subscale of the Hospital Anxiety and Depression Scale*.

### The Improved HRQoL in Patients Using FOB After SPT Interventions

**Figure 2:**
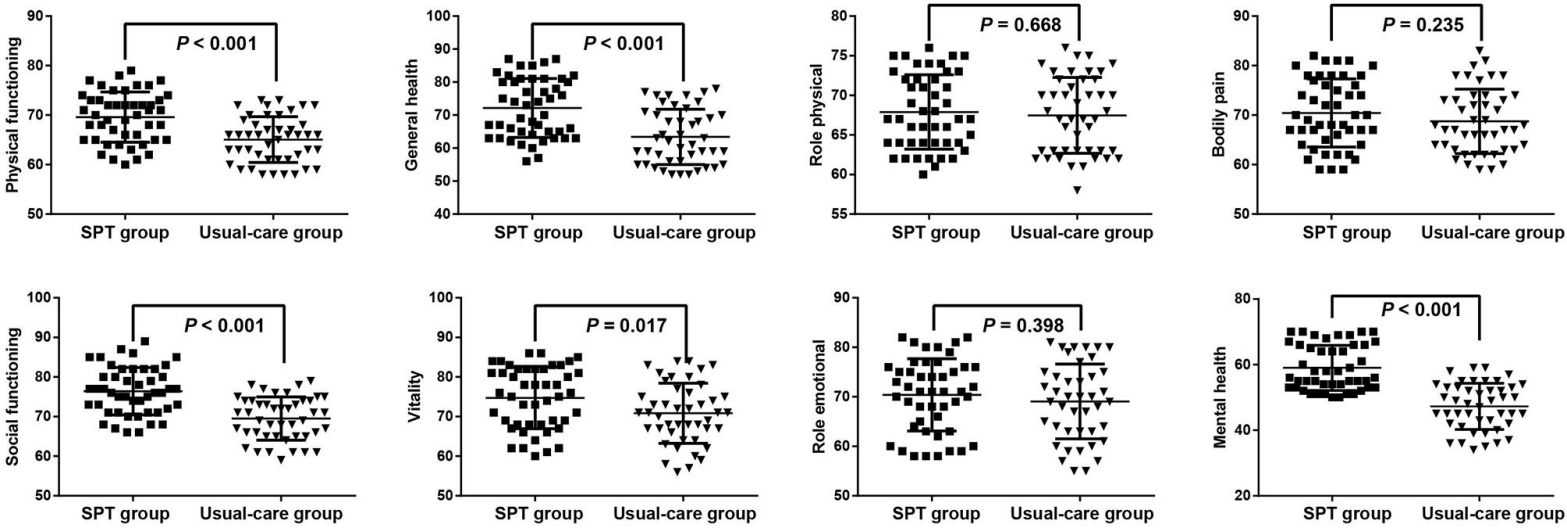
The alleviated improved health-related quality of life (HRQoL) in patients undergoing FOB after SPT.

Using SF-36 to measure the HRQoL of patients, the improved physical function (69.6 ± 5.08 vs. 65.07 ± 4.62, *P* < 0.001), global health (72.17 ± 8.95 vs. 63.4 ± 8.35, *P* < 0.001), social function (76.4 ± 5.97 vs. 69.51 ± 5.45, *P* < 0.001), vitality (74.74 ± 7.8 vs. 70.84 ± 7.61, *P* = 0.017), and mental health (59 ± 6.87 vs. 47.29 ± 7.06, *P* < 0.001) was found in the patients undergoing FOB after SPT interventions as compared to those received conventional nursing care (Table 4, Figure 2). However, the baseline score of SF-36 showed no significance between these two groups (*P* = 0.225). Moreover, highly HRQoL of patients after SPT interventions was revealed in relative to those at baseline, including physical function (69.6 ± 5.08 vs. 62.49 ± 4.9, *P* < 0.001), global health (72.17 ± 8.95 vs. 62.02 ± 7.79, *P* < 0.001), role physical (67.89 ± 4.71 vs. 65.81 ± 4.67, *P* = 0.034), social function (76.40 ± 5.97 vs. 66.47 ± 5.32, *P* < 0.001), vitality (74.74 ± 7.8 vs. 66.87 ± 7.66, *P* < 0.001), and mental health (59 ± 6.87 vs. 50.3 ± 6.79, *P* < 0001).

**Table 4:** Comparison of the health-related quality of life (HRQoL) using SF-36 in patients undergoing FOB.

**Parameters**	**SPT (*n* = 47)**	**Usual-care group** **(*n* = 45)**	**t**	***P***
Physical function				
Baseline	62.49 ± 4.90	62.80 ± 4.30	0.323	0.748
After intervention	69.60 ± 5.08	65.07 ± 4.62	4.468	<0.001
t	6.906	2.408		
*P*	<0.001	0.018		
Global health				
Baseline	62.02 ± 7.79	61.49 ± 8.38	0.316	0.753
After intervention	72.17 ± 8.95	63.40 ± 8.35	4.853	<0.001
t	5.863	1.084		
*P*	<0.001	0.282		
Role physical				
Baseline	65.81 ± 4.67	66.04 ± 4.79	0.239	0.811
After intervention	67.89 ± 4.71	67.47 ± 4.80	0.431	0.668
t	2.156	1.408		
*P*	0.034	0.163		
Bodily pain				
Baseline	68.49 ± 6.71	67.56 ± 6.25	0.690	0.492
After intervention	70.43 ± 6.88	68.76 ± 6.49	1.197	0.235
t	1.381	0.894		
*P*	0.171	0.374		
Social function				
Baseline	66.47 ± 5.32	67.62 ± 5.48	1.025	0.308
After intervention	76.40 ± 5.97	69.51 ± 5.45	5.774	<0.001
t	8.516	1.639		
*P*	<0.001	0.105		
Vitality				
Baseline	66.87 ± 7.66	68.51 ± 7.46	1.038	0.302
After intervention	74.74 ± 7.80	70.84 ± 7.61	2.426	0.017
t	4.935	1.469		
*P*	<0.001	0.146		
Role emotional				
Baseline	68.30 ± 7.31	67.93 ± 7.52	0.235	0.814
After intervention	70.38 ± 7.30	69.07 ± 7.57	0.849	0.398
t	1.383	0.712		
*P*	0.170	0.478		
Mental health				
Baseline	50.30 ± 6.79	48.67 ± 5.97	1.221	0.225
After intervention	59.00 ± 6.87	47.29 ± 7.06	8.063	<0.001
t	6.173	0.999		
*P*	<0.001	0.320		

*SPT, Supportive psychotherapy; SF-36, 36-Item Short Form Health Survey questionnaire; FOB, Fiberoptic bronchoscope*.

### Comparison of Satisfaction and Complication in Patients Undergoing FOB After SPT Interventions

**Figure 3:**
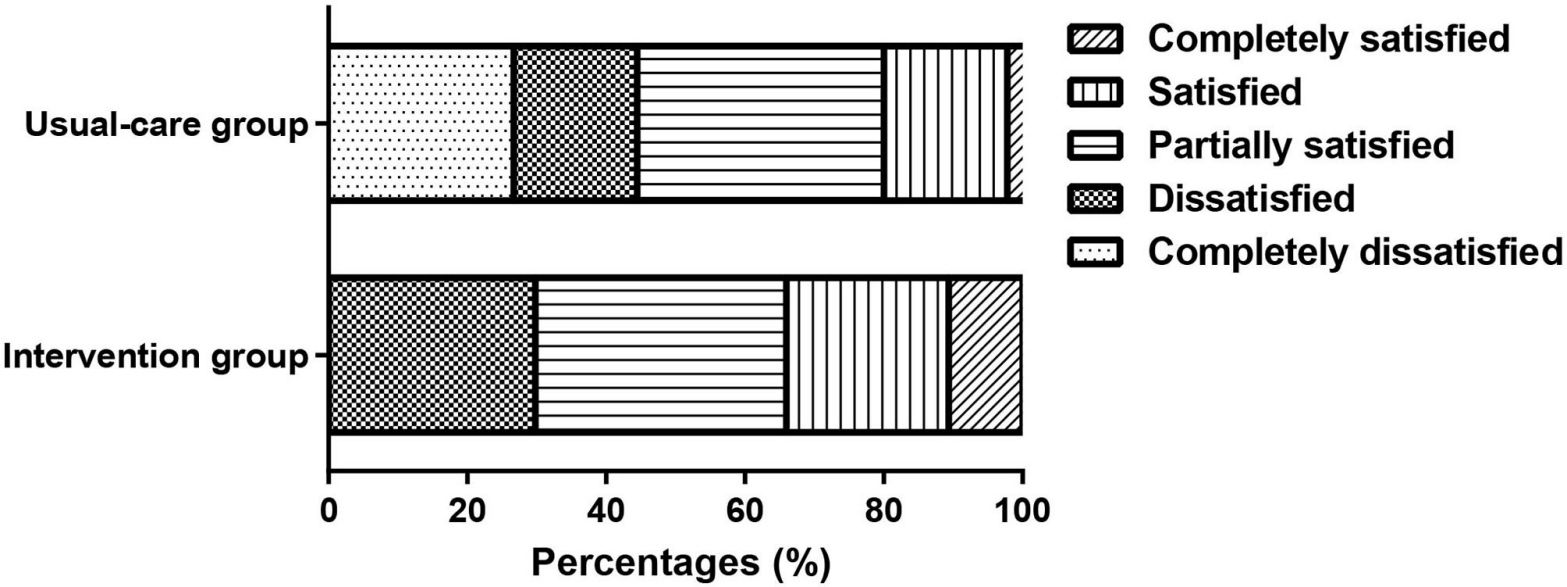
The percentages of completely dissatisfied, dissatisfied, partially satisfied, satisfied, and completely satisfied in the intervention and usual-care groups.

The patients' satisfaction was assessed based on the Likert 5-Point Scale, and the patients in the SPT group (3.15 ± 0.98) showed higher scores than the usual-care group (2.51 ± 1.14, *P* = 0.005). The percentages of completely dissatisfied, dissatisfied, partially satisfied, satisfied, and completely satisfied in the SPT group were 0, 29.79, 36.17, 23.4, and 10.64%, respectively, while the percentages in the usual-care group were 26.67, 17.78, 35.56, 17.78, and 2.22%, respectively (Figure 3). Moreover, the rate of complications was 6.38% in the SPT group (one patient with hypoxemia, and 2 patients with arrhythmias) and 11.11% in the usual-care group (1 case of hemoptysis, 1 case of hypoxemia, 1 case of fever, and 2 cases of arrhythmia), showing no significant difference.

## Discussion

Nurses are recognized as key persons who can help patients mitigate their negative thoughts and emotions, among which depression and anxiety are common reactions, and patients' emotional needs should be addressed during nurse counseling (Rutledge et al., 2018; Tay et al., 2018). SPT intervention, as an alternative method for giving patients self-confidence in their self-care practice, in understanding their problems, maintaining their hope, and reducing their anxiety (Ruchiwit, 2012), has been successfully used as a supportive treatment in many diseases, including hematologic malignancies (Koehler et al., 2022), body dysmorphic disorder (Weingarden et al., 2021), obesity (Juchacz et al., 2021), chronic back pain (Rutledge et al., 2018), and miscarriage and so on (Barat et al., 2020). However, there is a lack of evidence on its role in the management of patients undergoing FOB. In this study, we included the patients undergoing FOB in our hospital, which were divided into the SPT group and the usual-care group, and the patients in the two groups were paired according to age, gender, weight, ASA status, and Mallampati classification.

Screening for depression refers to the use of depression screening questionnaires to identify patients who may have depression (Levis et al., 2019). PHQ-9 and HADS-D, as the depression screeners designed for primary care, are the most commonly used screening tools for depression according to the many previous pieces of research (Cameron et al., 2008; Kendel et al., 2010; Reddy et al., 2010). Moreover, the HADS-A was developed to help identify anxiety disorders in people to avoid overlap with physical disorders (Wu et al., 2021). In our study, both PHQ-9 score, HADS-D score, and HADS-A score in the SPT group after SPT were lower than that in the usual-care group. In addition, in terms of the SPT group, the alleviated depression and anxiety were shown after the SPT intervention as compared with the baseline value, indicating the SPT had a good effect on the adverse emotions in patients undergoing FOB. Similarly, a previous also discovered a significant reduction in depression, anxiety, and stress scores of cancer patients who received SPT with an improved quality of life at six months (Mahendran et al., 2015). Besides, patients with planned first coronary artery bypass grafting showed significantly attenuated anxiety after SPT, suggesting the short-term SPT had a beneficial effect on reducing preoperative and postoperative anxiety, which was better than usual care (Heilmann et al., 2016). Ashman et al. reported that SPT was efficacious in improving diagnoses of depression and anxiety and reducing depressive symptoms in patients with traumatic brain injury (Ashman et al., 2014). Moreover, the improved HRQoL and the increased patient satisfaction were found in the patients undergoing FOB after SPT interventions as compared to those receiving conventional nursing care. Being consistent with our result, after SPT, there was a significant improvement in physical health, psychological health, environmental condition, and social relationship in female cancer patients receiving chemotherapy (Mukherjee et al., 2017), being consistent with our study. All mentioned above indicated that SPT interventions should be encouraged along with FOB to promote positive mental health and to obtain the full benefit of their physical treatment. However, there existed a few limitations in this study which were as follows: (1) sample size would be expanded to validate our result; (2) more clinical efficacy regarding the SPT interventions in patients undergoing FOB would be evaluated in the future; (3) the patient enrolled in the study had what kind of disease should be further considered.

Both PHQ-9 and HADS-D scores in the SPT group after SPT were lower than that in the usual-care group, accompanied by the deceased HADS-A subscale and the improved mental health. Additionally, the patient satisfaction in the SPT group was higher than in the usual-care group. These results advocate training for nurses and physicians to provide SPT to patients before, during, and after the FOB procedure.

## Data Availability Statement

The original contributions presented in the study are included in the article/supplementary material, further inquiries can be directed to the corresponding author.

## Ethics Statement

The studies involving human participants were reviewed and approved by Wuhan Wuchang Hospital. The patients/participants provided their written informed consent to participate in this study.

## Author Contributions

FJR, DR, and WLH contributed to the conception and design of research. WLH, YX, and YWW collected and analyzed data. YWW and DR interpreted results of experiments. YX and SYH prepared figures. DR, YWW, and YX drafted manuscript. FJR, SYH, and WLH edited and revised manuscript. All authors approved the final version of the manuscript.

## Conflict of Interest

The authors declare that the research was conducted in the absence of any commercial or financial relationships that could be construed as a potential conflict of interest.

## Publisher's Note

All claims expressed in this article are solely those of the authors and do not necessarily represent those of their affiliated organizations, or those of the publisher, the editors and the reviewers. Any product that may be evaluated in this article, or claim that may be made by its manufacturer, is not guaranteed or endorsed by the publisher.
